# Complex Probiotics Relieve Constipation Through Regulation of the Intestinal Microbiota in Kittens

**DOI:** 10.3390/microorganisms13030563

**Published:** 2025-03-01

**Authors:** Shimin Zhu, Zhengrong Guo, Lin Liu, Yuan Gao, Lu Bai, Yongfu Chen, Musu Zha

**Affiliations:** 1 Key Laboratory of Dairy Biotechnology and Engineering, Ministry of Education, Inner Mongolia Agricultural University, Hohhot 010018, China; 13603576436@163.com (S.Z.); 13451344075@163.com (Z.G.); yzwdmsnlinlin@163.com (L.L.); gy230218@126.com (Y.G.); bluu188@163.com (L.B.); nmgyfchen@126.com (Y.C.); 2 Inner Mongolia Key Laboratory of Dairy Biotechnology and Engineering, Inner Mongolia Agricultural University, Hohhot 010018, China

**Keywords:** kittens, probiotics, intestinal microbiome, constipation, health

## Abstract

The early developmental phase is a critical window for feline growth, during which immature digestive systems are susceptible to microbiome imbalances caused by environmental stressors. Our research employed macrogenomic analysis to evaluate how complex probiotic formulations influence growth metrics and gastrointestinal flora in juvenile felines. Two dozen healthy kittens were equally divided into the control group and the probiotics group following a 5-day environmental adaptation phase. Fecal scores were recorded daily for all kittens. Fresh fecal samples were collected on days 1 and 14 for macrogenomic analysis. The results showed a significantly lower rate of constipation in the probiotics group compared to the control group (*p* < 0.05). However, no significant differences were observed in intestinal microbial diversity or structure between the two groups. Metagenomic analysis revealed a higher relative abundance of *Bifidobacterium animalis* in the probiotics group compared to the control group (*p* < 0.05). Additionally, the probiotics group exhibited lower relative abundances of *Lachnospiraceae bacterium 2 1 58FAA*, *Lachnospiraceae bacterium 1 1 57FAA*, and *Acidaminococcus intestini* compared to the control group (*p* < 0.05). These results suggest that complex probiotics can regulate the intestinal microbiota, improve constipation, and promote intestinal health in kittens.

## 1. Introduction

A consistent upward trajectory characterizes global domestic cat populations, as evidenced by census data indicating 48.6 million registered felines in China, 95.6 million in US households, and 129.11 million across European Union territories [1]. Pets (mainly cats and dogs) are cared for and kept as companion animals to provide social support and companionship to humans. At the same time, cats and dogs are often considered more than just household pets; they are respected family members. Over the years, pet numbers have grown dramatically, as has interest in their health and well-being. Cats often experience stressful situations such as changes in seasons and temperatures, changes in the housing environment, and changing to different cat foods. This stress affects the cat’s health and may lead to diarrhea, loss of appetite, decreased disease resistance and depression [2]. Statistically, a high mortality rate is associated with diarrheal diseases of kittens in animal shelters in the United States and elsewhere [3]. Many kittens die of diarrhea before 8 weeks of age. The causes of diarrhea can be multifactorial, including immature immune systems, weaning stress, independent feeding, changes in food type, weakened maternal antibodies, and increased exposure to diarrheal pathogens. Due to the large number and variety of potential pathogens (viruses, bacteria, parasites, and protozoa), kittens without diarrhea often carry pathogens, hindering specific treatment [4]. Diarrhea in cats housed in animal shelters can delay the adoption process and deplete limited shelter resources. In extreme cases, it can lead to the euthanasia of the animal [5].

The presence of a wide range of complex microorganisms in the intestine is characteristic of all animals [6]. The balance of intestinal microbiota and their interactions with the host have strong impacts on the overall health of the host. The intestinal microbiota and health of pets are closely related. Intestinal microbiota are involved in the digestion, absorption, and metabolism of nutrients by the host. In terms of nutrient metabolism, intestinal microbiota play an important role in regulating various metabolic pathways, including metabolism of lipids, carbohydrates, and proteins. The composition and status of intestinal microbiota are closely related to the host’s intestinal health. Disturbances of the host’s intestinal microbiota may lead to various systemic diseases in the host, including gastrointestinal diseases, metabolic diseases, and obesity [7].

Probiotics can provide health benefits to the host when consumed in sufficiently adequate amounts [8]. Probiotics have a variety of beneficial effects such as inhibiting pathogenic bacteria colonization, maintaining intestinal microbiota homeostasis, promoting nutrient digestion and absorption, and preventing stress and diarrhea in animals. In recent years, probiotics have been widely used for the prevention and treatment of infectious diseases. In veterinary medicine, evidence shows that certain probiotics are beneficial for the treatment of acute and chronic diarrhea and inflammatory bowel disease [9]. Preliminary studies have been conducted on probiotics in cats; for example, *Enterococcus faecium* strain SF68 was shown to reduce the duration of diarrhea, maintain fecal microbial diversity in stressed cats and improve immunomodulation in cats [5,9]. The probiotic strain *Lactobacillus acidophilus* D2/CSL (CECT4529) improved fecal quality parameters in adult healthy cats [6]. A *Bacillus licheniformis* fermentation product relieved chronic diarrhea and improved the fecal microbiota of cats [8]. Complex *Bacillus* sp. has potential as a dietary supplement to alleviate diarrhea in pet cats [10]. *Lactobacilli* has a positive effect on the intestinal microbiota of cats, reducing odorous substances and improving the digestibility of nutrients [11]. Multi-strain probiotics promote intestinal health in cats by modulating the intestinal microbiota, improving microbiota-derived short-chain fatty acid production, reducing inflammation, and improving antioxidant status [7]. Probiotic and enzyme complexes improve the health of cats by reducing diarrhea incidence, decreasing the proportion of harmful bacteria in the intestine, improving the structure of the intestinal microbiota, and enhancing immunity [12]. *Bacillus* spp. probiotics improve the health of cats by reducing diarrhea, increasing the digestibility of nutrients, and providing antioxidant function [2].

Preliminary studies have been conducted on the effects of probiotics on cats. However, these studies have generally focused on adult cats, and few studies have explored the effects of probiotics on the intestinal tracts of kittens. Therefore, the aim of this study was to explore the effect of complex probiotics on the intestinal flora of kittens and to provide a basis for research on probiotic supplementation for kittens.

## 2. Materials and Methods

### 2.1. Animals and Experimental Design

Twenty-four healthy kittens (3–4 months old; 16 females and 8 males) were randomly allocated to control and probiotics groups based on their initial weight and sex. There was no significant difference in the mean baseline weights between the two groups of kittens (*p* < 0.05). We carried out the formal experiment for 14 days, after all kittens had acclimatized for 5 days. Each morning, we weighed the cat food remaining in each kitten’s food bowl as well as the fresh cat food supplied (C3 PEPTIDO milk cake cat food; Partido (Linyi) International Biotechnology Co., Ltd., Linyi, Shandong, China [13]). We calculated the daily intake of each kitten based on these data. In the afternoon of each day, we would clean the litter trays of all kittens. During this time, we assessed each kitten’s fecal score (FS) according to the Purina Fecal Score Scale. We considered a FS < 2 to indicate that a kitten is constipated. A FS > 4 indicates that the kitten has diarrhea. We weighed each kitten on days 1, 7, and 14 of the experiment. We calculated the weight change of each kitten based on these data. During the formal experiment, we fed kittens from the probiotics group a preparation of complex probiotics (*Bifidobacterium animalis* subsp. *lactis* BX-259, *Lactiplantibacillus plantarum* LP-301, and *Lacticaseibacillus rhamnosus* LR-78 mixed in a 1:1:1 ratio, 1.5 × 10^9^ colony-forming units [CFU]/d/kitten, Inner Mongolia Agricultural University, Hohhot, China) every morning. On days 1 and 14 of the experiment, we put 2 g of fresh feces from each kitten into sterile EP tubes. Afterwards, we stored the EP tubes immediately at −80 °C for macrogenomic analysis.

Before the start of the experiment, we immunized and dewormed all kittens as necessary. We made sure that the kittens had not taken any medication (e.g., antibiotics) that could cause changes in the intestinal microbiota 1 month prior to the start of the experiment. We placed each kitten in a separate cage (50 × 32 × 38 cm^3^). Each cage included a litter tray and food bowl. We placed all cages containing kittens in a large, airtight, windowed house to ensure that all kittens were subjected to the same environmental factors (e.g., temperature, humidity, etc.), which would not affect the experimental results. We provided all kittens with the same diet and plentiful clean drinking water. All kittens were provided with a variety of toys and scratching poles for environmental enrichment and human interaction. All cages were sterilized and cleaned regularly. At the end of the experiment, the kittens were not euthanized. All kittens were adopted to private homes. Our study strictly followed the requirements of the Guidelines for the Use and Care of Laboratory Animals and was approved by the Laboratory Animal Welfare and Ethics Committee of the Inner Mongolia Agricultural University (No. NND2023098).

### 2.2. Intestinal Microbiome Metagenomic Sequencing

DNA was extracted from fecal samples using the QIAamp Stool Mini kit (Novogene Co., Ltd., Chaoyang District, Beijing, China) following the manufacturer’s instructions. The quality of the extracted DNA was assessed according to the provided QC report. The extracted DNA was used as input material for library preparation. Briefly, genomic DNA was fragmented by sonication to an average size of 350 bp. The fragmented DNA was then end-polished, A-tailed, and ligated with full-length adapters compatible with Illumina sequencing. This was followed by PCR amplification to enrich the adapter-ligated DNA fragments. The PCR products were purified and the quality of the libraries was assessed and quantified using quantitative PCR (qPCR). The libraries meeting the quality standards were pooled based on their effective concentration and the required data volume. The pooled libraries were sequenced on the Illumina platform using the PE150 (paired-end 150 bp) strategy. Raw sequencing data in the FASTQ format were preprocessed using fastp (https://github.com/OpenGene/fastp, accessed on 30 November 2023.) to remove low-quality reads and obtain clean data. Quality control and de-hosting steps were performed to eliminate potential contaminants. The cleaned macrogenomic data were then classified and annotated using the HUMAnN2 pipeline for downstream analysis.

### 2.3. Statistical Analysis

We analyzed differences in the microbial communities between the two groups using the Wilcoxon rank sum test. All experimental data were expressed as mean ± standard error of the mean (SEM). *p* < 0.05 was considered statistically significant. Statistical analyses were performed using R (v.4.3.2). Alpha diversity was assessed using the Shannon and Simpson indices. Images were generated using the R package. β-diversity was assessed with principal coordinate analysis (PCoA). PCoA plots and structural analysis plots of fecal microbial communities were generated using TUTOOL (http://cloudtutu.com.cn/, accessed on 15 October 2024). Correlation heat maps were created using OmicStudio (https://www.omicstudio.cn/home, accessed on 15 October 2024).

## 3. Results

### 3.1. Effects of Complex Probiotics on Growth Performance

**Figure 1:**
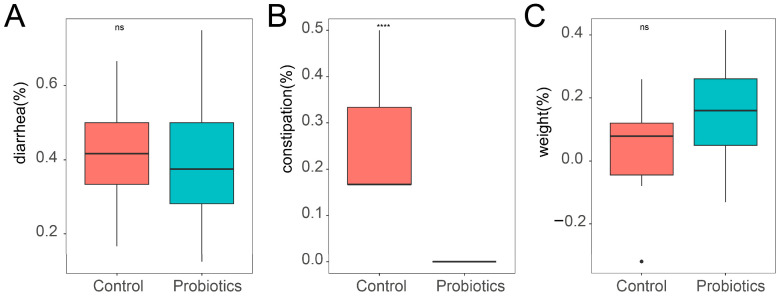
Effect of complex probiotics on the growth performance of kittens (*n* = 12). (**A**) Comparison of diarrhea incidence in kittens in the probiotic and control groups. (**B**) Comparison of constipation incidence between kittens in the probiotic and control groups. (**C**) Comparison of the rate of body weight change in kittens in the probiotic and control groups. Data are expressed as mean ± SEM. Differences are indicated by ns (*p* > 0.05), **** (*p* < 0.0001).

Our complex probiotics were well tolerated by the kittens in the probiotics group, with no feeding difficulties observed. We found that the diarrhea incidence of kittens in the probiotics group tended to be lower than in the control group, but this difference was not significant (Figure 1A, *p* > 0.05). None of the kittens in the probiotics group developed constipation, which was a significant difference compared to the control kittens (Figure 1B, *p* < 0.05). In terms of the rate of change in body weight, kittens in the probiotics group tended to have a lower rate of change compared to kittens in the control group, but this difference was not significant (Figure 1C, *p* > 0.05). These findings suggest that our complex probiotics can relieve constipation in kittens.

### 3.2. Effect of Complex Probiotics on the Fecal Metagenome

**Figure 2:**
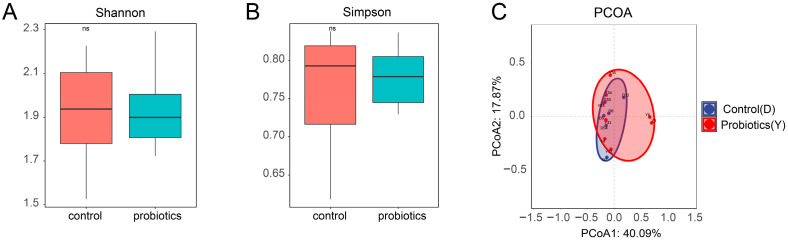
Diversity and structure of intestinal microbiota in kittens (*n* = 12). (**A**) Shannon index. (**B**) Simpson index. (**C**) PCoA plot. Data are expressed as mean ± SEM. Differences are indicated by ns (*p* > 0.05).

To investigate the effect of complex probiotics on the microbial diversity and structure of the intestinal microbiota in kittens, we first assessed changes in α-diversity by means of the Shannon and Simpson indices. As shown in Figure 2A,B, no significant difference (*p* > 0.05) was found between the fecal samples from the control and probiotic groups. Next, we assessed changes in the intestinal microbiota structure of kittens by means of PCoA plots. PCoA reflects information from samples in the form of points on a two-dimensional plane. Small sample intervals indicate that species have similar compositional structures. Thus, samples with high community structural similarity tend to cluster together, while samples with considerable community variation are placed far apart. Presented in Figure 2C, the PCoA plot shows no significant separation (*p* > 0.05) between fecal samples from the control and probiotic groups. Collectively, the α-diversity and β-diversity results indicate that no significant differences exist in the overall bacterial community diversity and structural composition between the probiotic and control groups.

**Figure 3:**
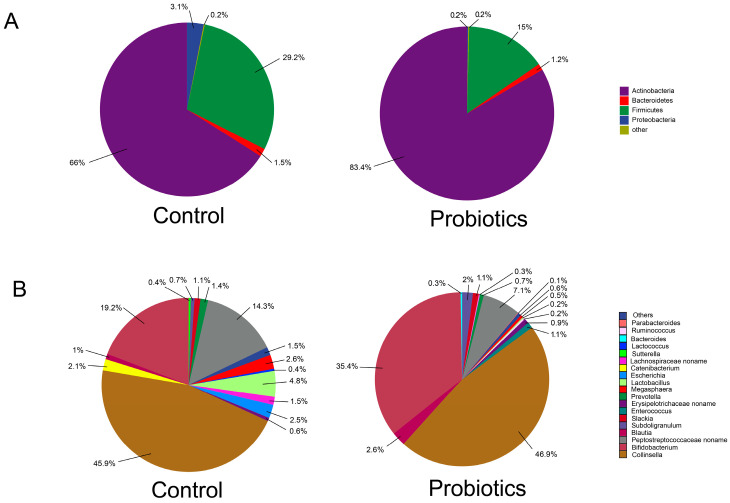
Composition of kitten intestinal microbiota at the phylum and genus levels (*n* = 12). (**A**) Relative abundance levels of major taxa at the phylum level in the control and probiotic groups on day 14. (**B**) Relative abundance levels of major taxa at the genus level in the control and probiotic groups on day 14.

The relative abundance levels of intestinal microbiota at the phylum level in the probiotic and control groups are shown in Figure 3A. The dominant phyla in the probiotics group were Actinobacteria (83.4%), Firmicutes (15.0%), Bacteroidetes (1.2%), and Proteobacteria (0.2%). The ratio of Firmicutes/Bacteriophages was 12.6. The dominant phyla in the control group were Actinobacteria (66.0%), Firmicutes (29.2%), Proteobacteria (3.1%), and Bacteriophages (1.5%). The ratio of Firmicutes/Bacteroidetes was 19.5. Treatment with complex probiotics reduced the ratio of Firmicutes/Bacteroidetes in the intestinal tracts of the kittens. The relative abundance of Actinobacteria and Firmicutes in both groups of kittens accounted for more than 90% of the total bacterial population.

The relative abundance levels of microbiota at the genus level in the probiotic and control groups are presented in Figure 3B. The top ten genera in terms of relative abundance in the probiotics group were *Collinsella* (46.9%), *Bifidobacterium* (35.4%), *Peptostreptococcaceae noname* (7.1%), *Blautia* (2.6%), *Subdoligranulum* (2.0%), *Slackia* (1.1%), *Enterococcus* (1.1%), *Erysipelotrichaceae noname* (0.9%), *Prevotella* (0.7%), and *Megasphaera* (0.5%). The top ten genera in terms of mean relative abundance in the control group were *Collinsella* (45.9%), *Bifidobacterium* (19.2%), *Peptostreptococcaceae noname* (14.3%), *Lactobacillus* (4.8%), *Megasphaera* (2.6%), *Escherichia* (2.5%), *Catenibacterium* (2.1%), *Lachnospiraceae noname* (1.5%), *Prevotella* (1.4%), and *Slackia* (1.1%). The same microorganisms were present with high relative abundance levels in the control and probiotic groups, namely *Collinsella*, *Bifidobacterium*, and *Peptostreptococcaceae noname*. No significant difference (*p* > 0.05) was found in the relative abundance levels of the top 10 microorganisms between the probiotic and control groups.

**Figure 4:**
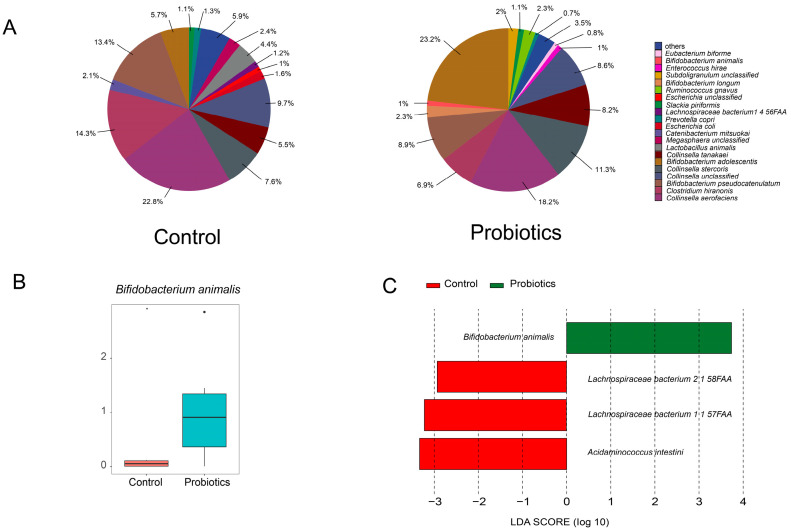
Composition and differences in the intestinal microbiota of kittens at the species level (*n* = 12). (**A**) Relative abundance levels of major taxa at the species level in the control and probiotic groups on day 14. (**B**) Comparison of the relative abundance of the species *Bifidobacterium animalis* between the probiotic and control groups on day 14. (**C**) Linear discriminant analysis Effect Size (LEfSe; linear discriminant analysis value [LDA] > 2.0). Data are expressed as mean ± SEM. * (*p* < 0.05) indicates statistically significant differences.

The relative abundance levels of major taxa at the species level in the probiotic and control groups are shown in Figure 4A. Comparison of the top fifteen microbial species in terms of relative abundance in the probiotic and control groups showed that only the relative abundance of *Bifidobacterium animalis* (*B. animalis*) differed significantly. The relative abundance of *B. animalis* was significantly elevated in the probiotics group compared to the control group (Figure 4B, *p* < 0.05). To explore further the effect of complex probiotics on the intestinal microbiota of kittens, we performed LEfSe analysis (LDA > 2.0) (Figure 4C). We found significant differences in the relative abundance levels of four bacterial species between the probiotics group and the control group. Complex probiotics significantly increased the relative abundance of *B. animalis* and decreased the relative abundance of *Lachnospiraceae bacterium 2 1 58FAA*, *Lachnospiraceae bacterium 1 1 57FAA*, and *Acidaminococcus intestini* (*A. intestini*) in the intestinal tracts of kittens. This result suggests that complex probiotics have a modulating effect on the intestinal microbiota of kittens.

### 3.3. Correlation Analysis

**Figure 5:**
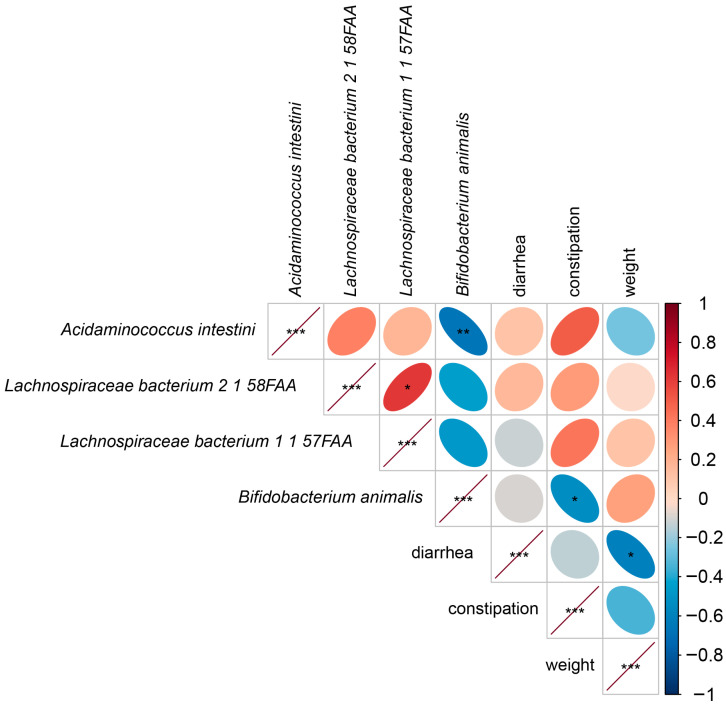
Spearman correlation analysis between intestinal microbiota and growth performance. Red represents positive correlations; blue represents negative correlations. The intensity of color reflects the strength of the correlation. All data are expressed as mean ± SEM. Significant differences are indicated by * (*p* < 0.05), ** (*p* < 0.01), and *** (*p* < 0.001).

To assess further the effect of the complex probiotics on the intestinal microbiota of kittens, we constructed a correlation coefficient plot based on Spearman correlation analysis (Figure 5). We found that *B. animalis* was significantly negatively correlated with constipation in kittens (R = −0.53, *p* < 0.05). *B. animalis* was also significantly negatively correlated with *A. intestini* (R = −0.67, *p* < 0.01). Diarrhea and weight were significantly negatively correlated (R = −0.6, *p* < 0.05). *Lachnospiraceae bacterium 2 1 58FAA* and *Lachnospiraceae bacterium 1 1 57FAA* were significantly positively correlated (R = −0.62, *p* < 0.05).

## 4. Discussion

As human society has developed, cats have become cherished companion animals and humans are becoming increasingly concerned about their health. Probiotics, which are widely used in pet cats, have numerous beneficial effects, including maintaining the stability of the intestinal microbiota, promoting the digestion and absorption of nutrients, and preventing stress and diarrhea in animals. However, studies to date have focused on adult cats, and few have been conducted on kittens. Therefore, the aim of this study was to explore the effect of complex probiotics on the intestinal health of kittens in terms of intestinal flora.

Kittenhood is a critical life stage for cats. During this period, kittens grow and develop rapidly and have a vigorous metabolism. However, the digestive system of kittens is not yet fully developed, resulting in weak intestinal absorption. Weaning is a common source of stress for kittens [14]. This stress can lead to disturbances in the intestinal microbiota, which can result in diarrhea and disease outbreaks. Probiotics have the ability to inhibit the colonization of pathogenic bacteria and maintain homeostasis of the intestinal microbiome. Some studies have reported that *Enterococcus faecium* SF68, *Bacillus* complexes, and fermentation products of *Bacillus licheniformis* alleviate diarrhea in cats [5,8,10]. However, in this study, we found no significant difference in diarrhea incidence between the probiotic and control groups of kittens. In our correlation analysis, we found a significant negative correlation between the rate of diarrhea and the rate of change in the body weight of kittens. This finding suggests that positive effects of complex probiotics on the rate of change in the body weight and the incidence of diarrhea in kittens occurred. Fecal scoring is a non-invasive and commonly used method to measure intestinal health and diarrhea in animals. Using fecal scoring, we found a significant reduction in the rate of constipation in kittens. These results suggest that complex probiotics improve the intestinal health of kittens by improving their fecal scores.

α-diversity is an indicator of the diversity of the intestinal microbiota, representing the richness and evenness of species within the sample. β-diversity is another diversity indicator, which represents the variability of communities between samples. In our experiments, there was no significant difference between the α-diversity and β-diversity of kittens in the probiotic and control groups. This finding is in accordance with the results of previous reports, as Li et al. [7] found that a multi-strain probiotic did not alter the diversity and structure of cats’ intestinal microbiota. Rossi et al. [15] observed that the multi-strain probiotic SLAB51TM also failed to change the diversity and structure of cats’ intestinal microbiota. Complex probiotics did not alter the diversity and structure of intestinal microbiota in kittens. This suggests that complex probiotics have a protective effect on the intestinal tract of kittens, therefore, they are an ideal treatment option [4].

Intestinal microbiota are biological barriers against colonization by pathogenic bacteria and are closely associated with host intestinal health. An imbalance of the intestinal microbiota may lead to host intestinal discomfort and can trigger the development of various diseases. Feline intestinal flora is similar to that of other mammals (including canines and humans) at the phylum level and consists mainly of Firmicutes, Bacteroidetes, Actinobacteria, and Proteobacteria [8,10,16]. In the present study, we observed decreases in the relative abundance of Firmicutes and Proteobacteria and an increase in the relative abundance of Actinobacteria in the probiotics group. Microorganisms associated with inflammation are present in the Firmicutes, whereas many health-promoting bacteria are included in phylum Bacteroidetes. Thus, the ratio of the Firmicutes/Bacteroidetes can be used to simply assess the health status of the host. We found that the ratio of the Firmicutes/Bacteroidetes was lower in the probiotics group than in the control group. This difference suggests that treatment with complex probiotics reduced the ratio of Firmicutes/Bacteroidetes in the intestinal tract of kittens, which may have a beneficial effect on intestinal health. Furthermore, studies have reported an increase in the abundance of Proteobacteria in cats with diarrhea [8]. The abundance of Proteobacteria was positively related to that of *E. coli* [10]. *E. coli* belongs to Proteobacteria and is closely associated with gastrointestinal disorders. In this study, we found that the relative abundance of Proteobacteria was lower in the probiotics group than in the control group. Thus, complex probiotics may have improved the intestinal health of kittens.

Based on LEfSe analysis, we found a significant increase in the relative abundance of *B. animalis* in the probiotics group. *B. animalis* is an important group of beneficial bacteria in the phylum Actinobacteria that reduces the level of coliform bacteria in feces and promotes gastrointestinal health [17,18]. Dogs subjected to psychological stress had reduced levels of *Clostridia* after administration of *B. animalis* [19]. Pang et al. [20] reported that *B. animalis* promotes the growth of weaned piglets by improving intestinal development, increasing the abundance of beneficial bacteria, and reducing the number of pathogenic bacteria. In this study, we found that *B. animalis* was significantly negatively correlated with *A. intestini* through correlation analysis. These results suggest that *B. animalis* promotes intestinal health in kittens by reducing the abundance of harmful bacteria. We also found a significant negative correlation between the constipation rate of kittens and *B. animalis*. *B. animalis* relieves acute diarrhea and produces optimal feces in dogs [17,18,19]. Therefore, *B. animalis* may promote intestinal health in kittens by improving fecal scores. We found an interesting phenomenon—that *B. animalis* was closely associated with intestinal health in animals, after which *B. animalis* could be further investigated as a key strain for intestinal health in kittens.

We detected significant decreases in the relative abundance of *A. intestini*, *Lachnospiraceae bacterium 2 1 58FAA*, and *Lachnospiraceae bacterium 1 1 57FAA* in the probiotics group. All three of these bacteria belong to the phylum Firmicutes. *A. intestini* is associated with inflammation and its abundance is significantly elevated in the intestinal microbiota of patients with cancer and depression [21,22,23]. *Lachnospiraceae bacterium 2 1 58FAA* and *Lachnospiraceae bacterium 1 1 57FAA* have been less thoroughly studied in animals. *Lachnospiraceae bacterium 2 1 58FAA* has high abundance in the intestinal tract of patients with recurrent inflammatory bowel disease [24]. One study found that peritoneal dialysis patients treated with the traditional herbal decoction Tiaopi Xiezhuo accumulated *Lachnospiraceae bacterium 2 1 58FAA* in their intestinal microbiota and experienced an improvement in constipation [25]. This finding contrasts with the results of our study. In the present study, kittens in the probiotics group had reduced levels of *Lachnospiraceae bacterium 2 1 58FAA* in the intestine and lower rates of constipation. In our correlation analysis, no significant correlation exists between *Lachnospiraceae bacterium 2 1 58FAA* and constipation incidence. Therefore, *Lachnospiraceae bacterium 2 1 58FAA* levels in the intestinal tracts of kittens were not associated with constipation. The relative abundance of *Lachnospiraceae bacterium 1 1 57FAA* was significantly higher in the feces of congested, depressed, and COVID-19 patients [26,27,28,29]. Our analysis showed a significant positive correlation between *Lachnospiraceae bacterium 2 1 58FAA* and *Lachnospiraceae bacterium 1 1 57FAA*. Therefore, although the literature did not indicate that *Lachnospiraceae bacterium 1 1 57FAA* is associated with intestinal inflammation, its correlation with *Lachnospiraceae bacterium 2 1 58FAA* suggests that it is similarly associated with intestinal inflammation in kittens. These results indicate that all three strains that were significantly reduced in the intestines of kittens after the administration of complex probiotics were associated with inflammation. Thus, complex probiotics improve intestinal health in kittens by reducing the risk of inflammation in the intestinal tract and thereby improving intestinal health.

**Figure 6:**
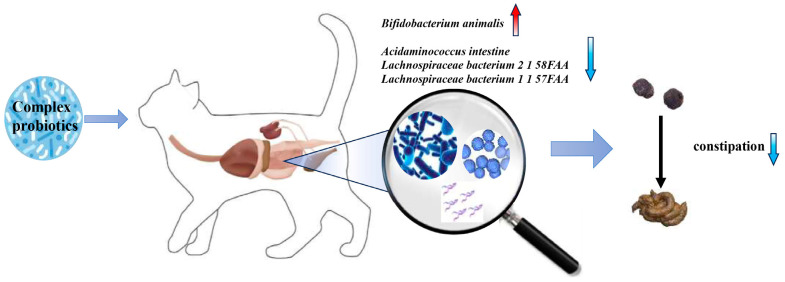
Simple mechanism of action of complex probiotics in the intestinal tract of kittens. Red represents an increase and blue indicates a decrease.

We summarize the mechanism of action of the complex probiotics in the intestinal tract of kittens (Figure 6). The complex probiotics alleviated constipation and promoted intestinal health in kittens by increasing the relative abundance of *B. animalis* and decreasing the relative abundance levels of *A. intestini*, *Lachnospiraceae bacterium 2 1 58FAA*, and *Lachnospiraceae bacterium 1 1 57FAA* in the intestines. This points us to the potential impact of complex probiotics on the intestinal microbiology of kittens. Further studies will be conducted to elucidate the specific mechanisms by which complex probiotics promote intestinal health in kittens.

## 5. Conclusions

This research demonstrates that complex probiotics can alter the intestinal microbiota of kittens. Specific microorganisms were associated with constipation and intestinal inflammation in kittens. Our results suggest that complex probiotics improve the intestinal health of kittens by regulating intestinal flora, improving fecal scores, and reducing the risk of inflammation. The present study suggests that complex probiotics have beneficial effects on kitten growth and have the potential for application as a nutritional supplement for kittens.

## Data Availability

The data generated from the study are clearly presented and discussed in the manuscript.
